# Learning to distinguish between predators and non-predators: understanding the critical role of diet cues and predator odours in generalisation

**DOI:** 10.1038/srep13918

**Published:** 2015-09-11

**Authors:** Matthew D. Mitchell, Douglas P. Chivers, Mark I. McCormick, Maud C.O. Ferrari

**Affiliations:** 1 Department of Biomedical Sciences, WCVM, University of Saskatchewan, SK, Canada; 2 Department of Biology, University of Saskatchewan, SK, Canada; 3 ARC Centre of Excellence for Coral Reef Studies, and College of Marine & Environmental Sciences, James Cook University, Townsville, QLD, Australia

## Abstract

It is critical for prey to recognise predators and distinguish predators from non-threatening species. Yet, we have little understanding of how prey develop effective predator recognition templates. Recent studies suggest that prey may actually learn key predator features which can be used to recognise novel species with similar characteristics. However, non-predators are sometimes mislabelled as predators when generalising recognition. Here, we conduct the first comprehensive investigation of how prey integrate information on predator odours and predator diet cues in generalisation, allowing them to discriminate between predators and non-predators. We taught lemon damselfish to recognise a predator fed a fish diet, and tested them for their response to the known predator and a series of novel predators (fed fish diet) and non-predators (fed squid diet) distributed across a phylogenetic gradient. Our findings show that damselfish distinguish between predators and non-predators when generalising recognition. Additional experiments revealed that generalised recognition did not result from recognition of predator odours or diet cues, but that damselfish based recognition on what they learned during the initial conditioning. Incorporating multiple sources of information enables prey to develop highly plastic and accurate recognition templates that will increase survival in patchy environments where they have little prior knowledge.

Detecting predators early is critical for prey, as it allows them to respond adaptively to risky situations and avoid costly and potentially life-threatening interactions with predators^1^,^2^. A pre-requisite for effective predator avoidance is that prey must first be able to recognise them and the threat they pose. Although some prey can innately recognise predators during their first encounter^3^,^4^,^5^, the spatial and temporal variability of predators means that prey must often learn about predators in their community. Learning allows prey to develop effective responses to predators as they gain experience but it comes at a cost^6^,^7^,^8^. To learn, prey must often survive an initial encounter with a predator^8^ and learning itself has fitness costs associated with the development and maintenance of neural structures involved in learning and memory^9^.

Recent advances in our understanding of how prey learn about predators has shown that learned recognition is not specific to a given predator species. Rather, recognition is based on some key features that can later be used to recognise novel predators based on similarities between novel species and the predators known to the prey^10^,^11^,^12^,^13^. For example, black-tailed deer (*Odocoileus hemionus columbianus*) recognised a model puma, their natural predator, as a threat and also responded to a model tiger, a non-native predator, with similar features to the puma but not to a model of a non-predatory mule deer or a leopard^14^. It was suggested that that lack of response to the leopard was due to its camouflaged spotted coat disrupting the body shape. Generalising predator recognition to novel species with similar features allows prey to gain innate-like recognition of unknown predators and reduces the costs associated with learning. However, two studies found that when using olfactory cues, both lemon damselfish (*Pomacentrus moluccensis*) and velvet geckos (*Oedura lesuerii*) were able to generalise recognition to novel related species but they were unable to distinguish between the odours of closely related predators and non-predators when generalising recognition^13^,^15^. The uncertainty associated with generalising recognition from a known predator to an unknown species means that, unless the traits used to generalise recognition are specific to predators, prey may potentially respond to non-threating species, resulting in a cost to their overall fitness. To account for errors resulting from uncertainty, it has been proposed that prey adjust their recognition templates in response to previous interactions with their local predator community and the level of threat posed by the known predators^10^. Alternatively, prey might be able to reduce uncertainty and make better informed decisions if they are able to use alternative sources of information relating to predator status^6^,^16^.

Along with chemical alarm cues (cues released from injured conspecifics that elicit innate anti-predator responses), diet cues are another important source of information used to label predators in both aquatic^17^ and terrestrial systems^5^,^18^. When a conspecific (or in some cases, heterospecific) gets consumed, an element of their chemical alarm cues survives the digestion process and acts much in the same way as chemical alarm cues would. These post-digestion alarm cues have been used by prey to instantly label predators through associative learning^19^,^20^ and learn about alarm cues from heterospecific prey guild members^21^,^22^. Yet, beyond their use as an unconditioned stimulus when they contain elements of alarm cues, the role of diet cues in risk assessment and predator recognition has been largely overlooked. Even in the absence of post-digestion alarm cues, diet cues have the potential to provide important information about predators, as predators with similar diets will likely overlap significantly in prey choice and foraging tactics. Diet cues potentially provide functional information that directly relates to the predatory status of a given animal. In the context of generalised recognition, diet cues could offer a second source of information that can be used to make better informed decisions about how to respond.

Our goal here was to provide the first comprehensive study of the effects of predator odours and diet cues in generalisation of predators and non-predators. We used three experiments to assess the interactive effect of information acquired about the predator (the predator’s signature odour) and information acquired about the predator’s diet (diet cues) on the pattern of generalisation of a reef fish living in a species diverse environment. Our previous study demonstrated that when diet cues were controlled for, juvenile lemon damselfish (*Pomacentrus moluccensis*) generalised recognition to other species within the same genus of a known predator. However, they did so irrespective of the actual predatory status of the congeners. This meant that the prey mislabelled some non-predatory congeners as predators. We suggested that such results reflected the threat-sensitive trade-off associated with correctly identifying predators in species-rich environments^13^. The early life-history of coral reef fishes consists of an initial dispersed larval stage followed by settlement onto coral reefs that are inhabited by a diverse community of opportunistic and specialist predators. Settlement mortality from predation is high (~60% on average over the first 2 days post settlement^23^) and in the absence of innate knowledge of predators^24^, prey are under extreme selection to identify non-predators from predators. Recent studies have shown that chemical cues play a critical role in predator recognition^13^,^24^,^25^,^26^ and survival at this point^27^,^28^,^29^. As such, our experiment directly replicates the predator-prey interactions being played out on coral reefs during this critical life-history transition period.

To achieve our overall aim we did a stepwise series of three experiments. To test the effect of diet cues on generalised recognition patterns, we conditioned juvenile lemon damselfish to recognise moon wrasse, *Thalassoma lunare*, which had been maintained on a piscivorous diet, as a threat. We then tested individuals for recognition of the odour of *T. lunare* itself or a selection of novel predators and non-predators, paired across a phylogenetic gradient. In this first study, fish were fed their natural diets, with predators maintained on a piscivorous diet matching that of the learned predator and non-predators maintained a non-fish (invertebrate) diet. Following this experiment, we ran two additional experiments to investigate the potential effects of species relatedness and diet cues on the pattern of generalisation seen. In a second experiment, by crossing diets cues and predator/non-predator cues, we tested whether generalisation patterns could be altered via the diet of the novel species. Finally, we investigated the role of the diet of the learned predator in the generalisation pattern, by conditioning damselfish with the odour of the predator fed one of two different diets. We predicted that diet cues might have one of three effects on the generalised predator recognition patterns: 1) Diet cues may have no effect on predator recognition – here we would expect to see recognition generalised to closely-related predators irrespective of diet, matching the results where diet cues are controlled for; 2) Diet cues form a component of recognition templates, but no specific information about the level of risk– prey might lack an innate recognition of specific diet cues but if diet cues are used to form predator recognition templates along with the predator odour itself, we would expect prey to generalise recognition to closely-related species that have been fed a similar diet to *T. lunare* but show a weaker or no response to closely-related species fed a different diet; 3) Diet cues may provide information on predation risk – prey may innately recognise the piscivorous diet as risky, and so we would expect the piscivorous diet cue to have either an additive or synergistic effect on learned responses and recognition to be extend to species beyond the constraints of phylogenetic relationship. We might also expect there to be some level recognition of the piscivorous diet irrespective of the initial conditioning regime.

## Results

### Experiment 1- Pattern of generalisation by prey exposed to novel species fed their natural diet

**Figure 1:**
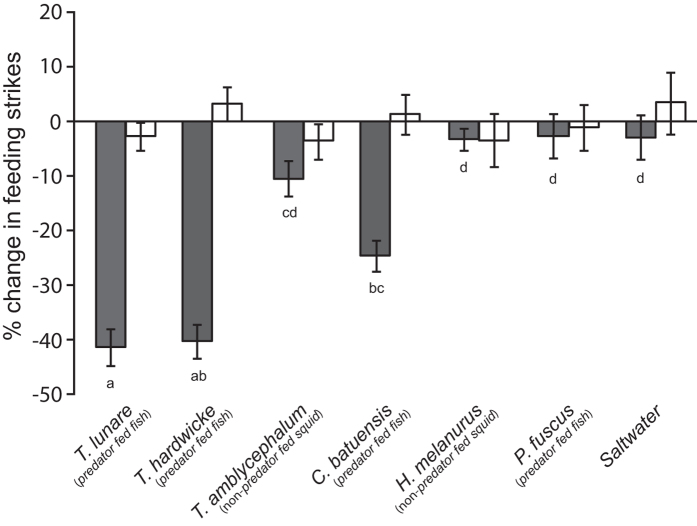
Pattern of generalisation by prey exposed to novel species fed their natural diet. Percent change in bite rate (per 5 min observation; mean ± 1 S.E.) for *Pomacentrus moluccensis* conditioned with the odour of *Thalassoma lunare* fed fish paired with a chemical alarm cue (grey bars) or saltwater (white bars) and tested for their response to the odours of predators and non-predators across a phylogenetic gradient or saltwater control. Predators were maintained on a fish diet and non-predators on a squid diet. Letters below bars indicate Tukey’s HSD groupings for fish conditioned with alarm cues.

Conditioning regime significantly affected the overall behavioural response to the test cues (MANOVA interaction: *F*_12,386_ = 4.7, *P* < 0.0001). Univariate ANOVAs showed there was a significant interaction between conditioning and test cues for feeding strikes (*F*_6,194_ = 14.3, *P* < 0.0001), but there was no effect of conditioning (*F*_1,194_ = 0.4, *P* = 0.54), test cues (*F*_6,194_ = 0.5, *P* = 0.83) and no interaction between the two factors (*F*_6,194_ = 0.3, *P* = 0.94) on line crosses. Individuals conditioned with *T. lunare* odour paired with alarm cue significantly reduced their feeding strikes following exposure to odours of *T. lunare* (*P* < 0.0001), *T. hardwicke* (*P* < 0.0001) or *C. batuensis* (*P* < 0.001) when compared to individuals conditioned with *T. lunare* odour paired with saltwater, indicative of an anti-predator response. Reductions in feeding strikes were similar between individuals exposed to *T. lunare* and *T. hardwicke* odours (*P* = 0.99) but individuals exposed to *C. batuensis* odours showed an intermediate response that was significantly weaker than those exposed to *T. lunare* odour (*P* < 0.05) but not *T. hardwicke* odour (*P* = 0.12). Individuals exposed to *T. amblycephalum*, *H. melanurus*, *P. fuscus* or the saltwater control did not alter feeding strikes irrespective of the conditioning regime (Fig. 1). These results demonstrate that *P. moluccensis* are able to generalise predator recognition to closely-related species that have consumed a similar diet but not those maintained on a different diet.

### Experiment 2 - Diet reversal in congeneric predator and non-predator species

**Figure 2:**
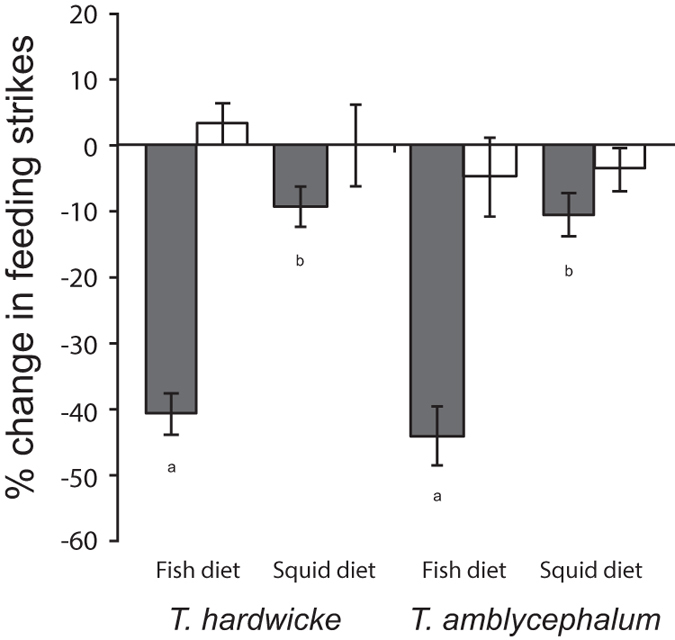
Diet reversal in congeneric predator and non-predator species. Proportional change in bite rate (per 5 min observation; mean ± 1 S.E.) for *Pomacentrus moluccensis* conditioned with the odour of *Thalassoma lunare* fed fish paired with an alarm cue (grey bars) or saltwater (white bars) exposed to the odours of a congeneric predator (*T. hardwicke*) or congeneric non-predators (*T. amblycephalum*) fed either fish or squid. Letters below bars indicate Tukey’s HSD groupings for fish conditioned with alarm cues.

The 3-way MANOVA revealed a significant interaction between conditioning and test species’ diet (*F*_2,108_ = 15.83, *P* < 0.0001), but no other interactions affected prey behaviour. Univariate ANOVAs showed that this interaction was significant for feeding strikes (*F*_1,116_ = 28.83, *P* < 0.0001) but not line crosses (*F*_1,116_ = 2.28, *P* = 0.13). Tukey’s HSD showed that individuals conditioned with alarm cue significantly reduced their feeding strikes when exposed to both *T. amblycephalum* (non-predator) and *T. hardwicke* (predator) maintained on the fish diet compared to individuals conditioned with saltwater (Fig. 2), but there was no difference in the intensity of the response between the two (*P* = 0.99). Feeding strikes for individuals exposed to *T. amblycephalum* (*P* = 0.98) and *T. hardwicke* (*P* = 0.65) fed squid did not differ between conditioning regimes, nor between the two treatments (*P* = 0.99). The univariate ANOVA for line crosses revealed that there was no effect of treatment (*F*_1,116_ = 2.24, *P* = 0.14), diet (*F*_1,116_ = 0.08, *P* = 0.78), conditioning (*F*_1,116_ = 0.01, *P* = 0.93) and no interactions between treatment and diet, treatment and conditioning, diet and conditioning (*F*_1,116_ = 2.28, *P* = 0.13) or treatment, diet and conditioning (*F*_1,116_ = 0.19, *P* = 0.67).

### Experiment 3- Generalisation pattern based on known predators’ diet

**Figure 3:**
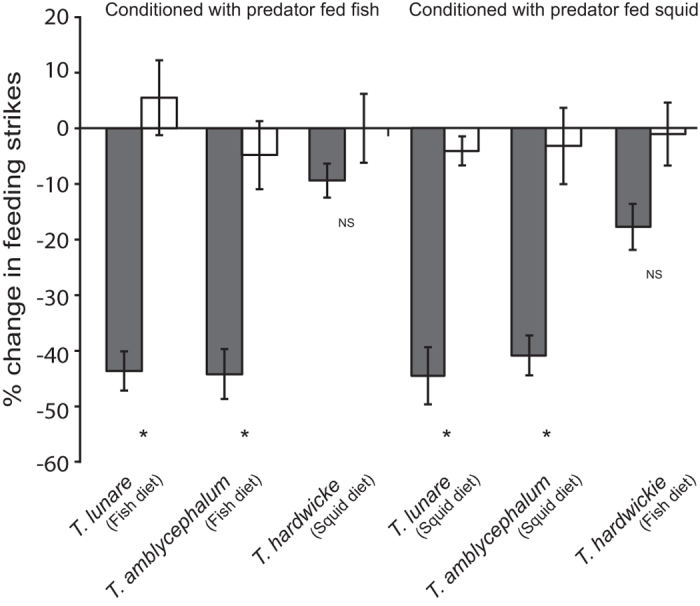
Generalisation pattern based on known predators’ diet. Proportional change in bite rate (per 5 min observation; mean ± 1 S.E.) for *Pomacentrus moluccensis* conditioned with the odour of *Thalassoma lunare* fed either fish or squid and paired with either chemical alarm cue (true conditioning; grey bars) or saltwater (false conditioning; white bars). Individuals were then exposed to the odours from *T. lunare* fed fish, *T. amblycephalum* or *T. hardwicke*. Predators and non-predators were fed either fish or squid depending on the treatment. Stars below bars indicate significant differences between true and false conditioning treatments.

There was a significant interaction between conditioning and predator diet on prey behaviour (MANOVA, Wilks, *F*_4,334_ = 7.03, *P* < 0.0001). The univariate ANOVA for feeding strikes showed a significant interaction between conditioning and predator diet (*F*_2,168_ = 10.59, *P* < 0.0001). There were no differences in the intensity of responses or how individuals generalised recognition between the two conditioned diets (fish or squid). Individuals that received the true conditioning (alarm cue) responded only to species that were fed the same diet as the learned predator. Individuals that received the false conditioning (saltwater) did not respond to any stimuli (Fig. 3). This suggests that diet is learned during the conditioning phase. The univariate ANOVA for line crosses revealed that there was no effect of conditioned diet (*F*_1,168_ = 3.87, *P* = 0.05), treatment (*F*_2,168_ = 1.99, *P* = 0.14), conditioning (*F*_1,168_ = 0.15, *P* = 0.70) and no interactions between conditioned diet and treatment (*F*_2,168_ = 0.76, *P* = 0.47), conditioned diet and conditioning (*F*_1,168_ = 2.26, *P* = 0.13), treatment and conditioning (*F*_2,168_ = 2.64, *P* = 0.07) or conditioned diet, treatment and conditioning (*F*_2,168_ = 0.47, *P* = 0.62).

## Discussion

Diet cues are known to play an important role in chemically-mediated predator recognition but researchers have generally only considered their role when they contain an element of an alarm cue^19^,^20^,^22^. The results from this study demonstrate that diet cues, lacking a known alarm cue component, play a previously unrecognised role in predator recognition. By incorporating diet cues into generalised recognition templates, prey are able to gain the benefits of innate-like predator recognition while reducing the costs of responding to non-threatening species. Consistent with previous studies investigating generalised recognition^10^,^13^,^14^,^30^,^31^, we found that damselfish conditioned to recognise *T. lunare* odour as a predation risk subsequently recognised *T. lunare* odour and also the odour of closely-related species as a threat. Recognition was not extended beyond the family level and the intensity of the anti-predator responses diminished with increasing phylogenetic distance between the known predator and the novel species. Our study, however, reveals that the inclusion of different diet cues among closely related species resulted in generalised recognition only to those species that were on the same diet as the learned predator (Experiment 2 and 3). Diet cues alone did not facilitate generalised recognition, since distantly-related *P. fuscus* was not recognised as a threat, despite sharing the same diet as *T. lunare* (Experiment 1). Their role seems to be integrated only when the novel species are closely related to the known predator. Response patterns were similar irrespective of *T. lunare’s* diet (squid or fish) during conditioning (Experiment 3); damselfish responded only to species on the same diet as *T. lunare*. This suggests that damselfish do not innately recognise a piscivorous diet as more risky than an invertebrate diet, but rather use diet cues to refine the recognition templates during the conditioning event.

Acquiring information about the local environment allows individuals to make better informed decisions about how to respond to future events^6^,^16^. Recently, Ferrari *et al.* (2007) suggested that the various mechanisms through which predator recognition arises is dependent on levels of uncertainty associated with predator cues in their local environment. Our results demonstrate that prey use two distinct sources of information available from predators to make more accurate decisions about how to respond to unknown species. Predator odours provide information on predator status based on the fact that closely-related species are often ecologically similar, and therefore share similar foraging ecology and diet preferences^10^. Diet cues, on the other hand, provide functional information about a predator (whether it is piscivorous or not), irrespective of phylogenetic relationships. Predators that produce similar diet cues target similar prey but may have quite different foraging ecologies. Incorporating both diet cues and phylogenetic information into generalised recognition templates allows prey to reduce uncertainty around the identity of novel species.

Our results provide an insight into how information from the two cues is processed and incorporated into recognition templates. As expected, generalised predator recognition was dependant on phylogenetic relationships, with only closely-related species being recognised as threatening. While diet cues altered the responses within this template, matching diet cues alone did not confer recognition beyond the limits of the phylogenetically-based template, supporting the predictions of previous studies^10^. This would suggest that recognition templates are primarily based on predator odour (i.e., the signature of the predator irrespective of its diet). Diet cues then provide supplementary information that fine-tunes the prey’s response pattern, with recognition being confirmed only when there is a positive match for both the phylogenetic relationship to the known predator, and the diet. This hierarchical cue preference reflects the relative reliability and consistency of the information provided by each of the two cue types through time. Predator signatures are stable over time scales at which selection occurs. Due to temporal and spatial variability in diet choice by predators, diet cues are less reliable in the information they provide.

The ability to distinguish between predators and non-predators did not result from prey associating the different diets as more or less risky. In contrast, studies on mammals have shown that prey can recognise predators through olfactory cues in urine and faeces^4^,^5^,^18^,^32^. For example, mice and rats have shown that they associate diet cues from mammalian predators with a greater level of risk compared to those from non-predatory herbivores^4^,^32^, due to 2-phenylethylamine found in the urine of all carnivorous species. We suggest that these apparently contradictory results can be explained if we consider the evolution of responses to diet cues in the context of the predator recognition continuum hypothesis^10^. Applying this theory to diet cues, innate responses should develop in environments where predator diversity is low and predators target more specific prey; that is, in systems where diet cues provide reliable information about risk such as the rodent/mammal system above. At this point, diet cues can act as an unconditioned stimulus allowing prey to learn predators based on diet cues alone or they may increase the overall level of risk during conditioning with another unconditioned stimulus resulting in prey generalising to a wider range of species, as seen in threat sensitive predator generalisation^31^. On the other hand, learning which diets are risky and generalising diet recognition should be beneficial when predators are diverse and target a wide selection of prey. In accordance with this hypothesis, we predict that in our system, diet cues do not act as a simple on/off switch as our results might suggest, but are generalised. Such a mechanism would allow prey to recognise predators with similar but not identical diets to the reference predator. Generalising both predator odour and diet cues would provide prey with a flexible mechanism for predator recognition through which threat-sensitive trade-offs can be optimised to increase survival and fitness during critical early encounters with predators. Given that a number of prey fish respond with a gradually weaker intensity to alarm cues released by increasingly distant relatives^33^,^34^, it is not unreasonable to suggest a similar pattern would occur, should those alarm cues be ingested, rather than released fresh in the water column.

If selection favours prey using diet cues as a means of reducing predation success via predator labelling, the flip side of the evolutionary arms race should also select for a predator’s ability to manipulate the prey’s perception of the predator’s diet. Indeed, predators might be able to influence how prey learn about them and respond to the threat they pose by varying their diets. When we also consider that diet cues can represent different levels of risk^35^, generalist predators that forage on a range of prey will provide different information about risk. Creating uncertainty should reduce the rate which prey learn to recognise them and lower the level of risk with which they are perceived. Conversely, specialist predators with restricted diets will be recognised rapidly and as a high risk. By targeting specific prey, predators may inadvertently increase both how conspicuous they are to prey and prey’s vigilance towards them, impairing their ability to forage efficiently. Optimal foraging theory predicts that the value of prey also decreases over time due to increased vigilance making prey harder to catch^36^. As a result, some predators are able to adjust the time at which they switch between foraging patches to account for prey vigilance^36^. Lima *et al.*^37^ recognised the importance of prey vigilance and incorporated it into classical models of optimal foraging and showed that due to increased levels of prey vigilance, predators should switch to more generalist diets than expected. Furthermore, the potential importance of diet cues in mediating predator-prey interactions is highlighted by various studies showing that predators will defecate away from their foraging grounds in order to lower prey vigilance^38^. Understanding how predators manage their prey’s anti-predator responses has important consequences for how predators optimise foraging. These results demonstrate that by using multiple cues prey are able to efficiently learn about predators in their local environment, and also suggest that there may be previously unrecognised benefits to predators when targeting a variety of prey.

## Methods

### Study species

**Figure 4:**
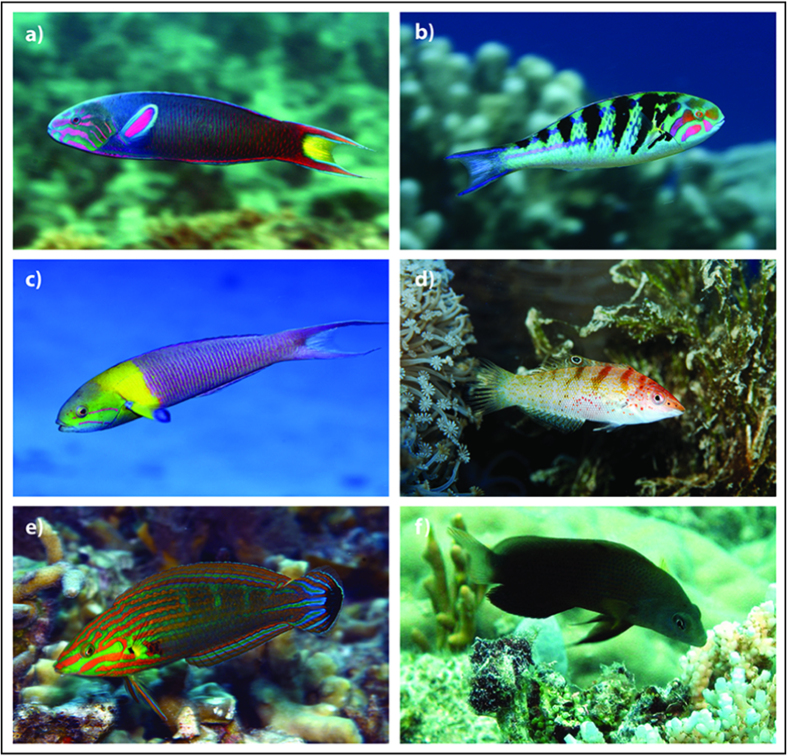
Predator and non-predator species. Images of (**a**) *Thalassoma lunar –* learned predator, (**b**) *Thalassoma hardwicke –* congeneric predator, (**c**) *Thalassoma amblycephalum –* congeneric non-predator, (**d**) *Coris batuensis –* confamilial predator, (**e**) *Halichoeres melanurus –* confamilial non-predator and (**f**) *Pseudochromis fuscus –* distantly related predator. Photos a *–* e copyright by Jeanette Johnson (© In-Depth Images Kwajalein). Retrieved 16 June 2015, http://www.underwaterkwaj.com/. Reproduced with permission of creator. Photo f courtesy of M.I.M.

Lemon damselfish are a common planktivorous, coral reef fish, found throughout the Indo-Pacific. Juvenile damselfish are consumed by a wide range of small opportunistic predators including wrasses such as the moon wrasse, *Thalassoma lunare*^39^. Wrasses (family, *Labridae*) are a diverse and abundant family that feed predominantly on small fishes and invertebrates, often switching opportunistically between the two^39^,^40^,^41^. We used moon wrasse as our learned predator, six-bar wrasse (*Thalasomma hardwicke*) as a congeneric predator, variegated wrasse (*Coris batuensis*) as a confamilial predator and brown dottyback (*Pseudochromis fuscus*) as a distantly-related predator^39^,^41^,^42^,^43^ (Fig. 4). All the predators in the study have a broadly similar ecology, spending most of their time associated with benthic coral reef habitats and foraging on juvenile fish recruits and benthic invertebrates. The congeneric non-predator, blunt -headed wrasse (*Thalassoma amblycephalum*) is found in the water column over the reef crest and reef slope where it feeds on planktonic invertebrates^32^. The confamilial tail-spot wrasse (*Halichoeres melanurus*) is found associated with the benthic coral reef habitat and feeds on benthic invertebrates^43^,^44^.

### Collection and maintenance

Fishes were collected from the fringing reef surrounding Lizard Island on the northern section of the Great Barrier Reef, Australia (14^0^ 40′S, 145^0^ 28′E). Settlement stage *P. moluccensis*, naïve to odours from reef-associated predators, were collected using light traps placed 50-100 m off the reef crest during November and December 2011 and 2012. Following collection, fish were transferred to 60-L flow-through maintenance tanks and fed twice daily with freshly hatched *Artemia sp*. (250 individuals per mL).

All fish species were collected from reefs at Lizard Island using barrier nets or clove oil, and maintained in aerated 40-L aquaria, with shelter. Fish were fed an approximately equal weight of either freshly-killed juvenile apogonids (*Apogonid sp*.) or squid depending on the experimental treatment. Juvenile apogonids (~20 mm total length) were collected live off the reef using hand nets, stored in 40-L aquaria and fed live *Artemia sp.* Before being fed to the fish, they were humanely euthanised in accordance with James Cook University Animal Ethics Guidelines (Protocol A1067). Juvenile apogonids are members of the same prey guild as damselfish (pers. obs.) but do not possess an alarm cue that elicits an anti-predator response in damselfishes. Thus they were selected to represent a diet that provides pertinent information about the identities of damselfish predators but lacks a recognised alarm cue component. The non-predators in this study predominantly target benthic invertebrates as their main food source. As collecting reliable quantities of such invertebrates was not feasible, we used bait squid to represent a low-risk invertebrate diet. Squid cues do not elicit damselfish anti-predator behaviour^13^,^24^.

### Stimulus preparation

The chemical alarm cues used during conditioning were prepared fresh for each round of conditioning. Fish were humanely sacrificed and 15 superficial cuts were made along each flank of each fish. The fish were then rinsed in 15 mL of seawater. This solution was then filtered to remove any solids. We used one fish per conditioning trial.

Predator and non-predator odours were prepared as per Mitchell *et al.* (2013). Briefly, fish were fed their experimental diet (4 individual juvenile apogonids or squid) for two days prior to collecting the odours from the storage aquaria. This ensured that any unknown gut content had passed though the fish. To produce the odour, the flow through system in each 40-L tank was turned off and water levels were set so that there was 1.1 g of fish per L of seawater. This was done to account for the different sizes of fishes and standardise the concentration of odours produced. Each aquarium was flushed every day at 1600 h by exchanging 80% of the water. Fishes were fed 1 h prior to the water exchange. Any uneaten food and faecal matter was removed during the water exchange. The aerated tanks were then left over night and the odours used the following morning.

### Observation tanks

Behavioural observations were conducted in 13-L flow-through aquaria (36 × 21 × 20 cm). Each aquarium contained a 1 cm layer of sand, a small shelter (5 cm diameter, 5 cm long terracotta pot) under the outflow and an air stone at the opposite end. Attached to the air stone was 1 m length of air tubing used for the introduction of food and the various chemical stimuli. A 4 × 6 grid was marked on the front of each tank to allow for behavioural measures and black plastic was placed around the other three sides to visually isolate each aquarium. For the duration of each trial, a black plastic curtain was hung in front of the aquaria to create an observational blind.

### Experiment 1- Pattern of generalisation by prey exposed to novel species fed their natural diet

This experiment followed a 2 × 7 design, where fish were taught, or not, to recognise *T. lunare* as a predator, and subsequently tested for their responses to *T. lunare* (learned predator), *T. hardwicke* (congeneric predator), *T. amblycephalum* (congeneric non-predator), *C. batuensis* (confamilial predator), *H. melanurus* (confamilial non-predator), *P. fuscus* (distantly-related predator) or a seawater control. The experiment was conducted in two stages, a conditioning stage and a recognition stage. All predators were fed the fish diet, while all the non-predators were fed the invertebrate diet. Diets were assigned to reflect the natural diets of the predators and non-predators. Individual *P. moluccensis* were placed in individual aquaria and allowed to acclimate for at least 2 h before the flow-through system was turned off for the conditioning stage. We then injected either 15 mL of alarm cue paired with 30 mL of *T. lunare* odour (true conditioning) or a non-learning control group exposed to 15 mL of saltwater paired with 30 mL of *T. lunare* odour. All stimuli injections were followed by 20 mL of water previously withdrawn from the tank to ensure that stimuli were completely flushed into the tank. After 1 h, the flow-through system was turned on to flush the odours out of the aquaria.

The following day, we conducted the recognition trials, which consisted of a 5-min pre-stimulus observation followed by a 5-min post-stimulus observation. Five min prior to the start of each trial, the flow-through system was turned off and 2.5 mL of food was injected into the tank. This allowed feeding rates to stabilise. At the start of the pre-observation period, a further 2.5 mL of food was injected into the aquarium and each fish was observed for a 5-min period. Once the pre-stimulus observation was completed, a further 2.5 mL of food were injected, followed by 30 mL of a randomly assigned stimulus odour (one of the 7 testing cues). The post-stimulus observation began directly after the injection of the stimulus odour. We conditioned a total of 210 damselfish with 12-15 replicates in the control treatments and 15-19 replicates for the experimental treatments.

During observation periods, we quantified two response variables: number of feeding strikes and number of line crosses. Decreased foraging and activity are established anti-predator responses in a number of prey species^45^. All feeding strikes were counted irrespective of whether they were successful or not. Line crosses were counted every time the entire body of the fish crossed a grid line.

### Experiment 2- Diet reversal in congeneric predator and non-predator species

Patterns of generalisation from Experiment 1 indicated that prey generalise their anti-predator response to closely-related predator species, but do not respond to non-predator species, regardless of their relatedness to the learned predators. This could be due to differences in the natural odour signature of the predators and non-predators, which could facilitate categorisation by the prey, or this pattern could simply be due to the difference in diet. To discriminate between these two alternatives, *P. moluccensis* were once again conditioned with alarm cue (or a water control) to recognise the odour of *T. lunare* fed a fish diet. They were subsequently tested for their response to *T. hardwicke* (congeneric predator) fed either fish or squid and *T. amblycephalum* (congeneric non-predator) fed either fish or squid. Data for this experiment were collected over 2011 and 2012. To control for any year effect, we contrasted baseline behaviour and learned response to *T. lunare* odour between the two years and found no difference between the years (fish from both years displayed similar intensities of anti-predator responses to their learned predator *T. lunare*) so the data were pooled. We conditioned a total of 119 damselfish with 13–17 replicates per treatments.

### Experiment 3- Generalisation pattern based on known predators’ diet

The results of Experiment 2 indicate that the generalisation pattern appears to be based on the diet of the novel species, and that differences in the chemical signatures of predators vs. non-predators (irrespective of diet) were not playing a role in the generalisation pattern. However, it was unknown if the prey were responding to novel species that were fed fish because they could recognise the piscivorous diet as risky, or whether the response was simply based on what the learned predator was fed. To distinguish between these two possibilities, we conditioned damselfish to the odour of *T. lunare* fed either fish or squid. Damselfish conditioned with odour from *T. lunare* fed fish were then tested for their response to odours of *T. lunare* fed fish, *T. amblycephalum* fed fish and *T. hardwicke* fed squid. Damselfish conditioned with odour from *T. lunare* fed squid were then tested for their response to odours of *T. lunare* fed squid, *T. amblycephalum* fed squid and *T. hardwicke* fed fish. This design allowed us to determine whether it was possible to elicit a fear response to the non-predator solely based on the match/mismatch between the novel species and the diet of the learned predator. We conditioned a total of 181 damselfish with 14–17 replicates per treatment.

### Ethics statement

Research was carried out under approval from the Great Barrier Reef Marine Park Authority and in accordance with James Cook University animal ethics guidelines, permit: A1067.

### Statistical analysis

For all experiments, we computed a proportional change in behaviour from the pre-stimulus baseline ((post-pre)/pre), which was used as the response variable in subsequent analyses. Data for foraging met the assumptions of homogeneity of variance and normality but data for line crosses needed to be log_10_ transformed to meet assumptions. Due to their lack of independence, the two response variables (feeding strikes and line crosses) were analysed simultaneously using a MANOVA approach.

#### Experiment 1

We tested the effect of conditioning (true vs. false conditioning) and test cue (7 cues) using a two-way MANOVA on the two response variables. Subsequent ANOVAs and Tukey’s HSD post-hoc comparisons were performed on individual variables to further investigate the response patterns.

#### Experiment 2

We ran a three-factor MANOVA to test the effect of conditioning (alarm cue vs. saltwater), test species (*T. amblycephalum* vs. *T. hardwicke*) and test species’ diet (fish vs. squid) on the responses of the prey. This analysis was, once again, followed by ANOVAs and Tukey’s HSD comparisons on individual variables.

#### Experiment 3

We ran a three-way MANOVA to test the effects of predator diet (*T. lunare* fed fish vs squid), conditioning (alarm cue vs. saltwater) and test species (*T. lunare* vs. *T. amblycephalum* vs. *T. hardwicke*). This was followed by ANOVAs and Tukey’s HSD comparisons on individual variables.

## Additional Information

**How to cite this article**: Mitchell, M. D. *et al.* Learning to distinguish between predators and non-predators: understanding the critical role of diet cues and predator odours in generalisation. *Sci. Rep.* **5**, 13918; doi: 10.1038/srep13918 (2015).
